# Full-genome sequencing and mutation analysis of SARS-CoV-2 isolated from Makassar, South Sulawesi, Indonesia

**DOI:** 10.7717/peerj.13522

**Published:** 2022-06-10

**Authors:** Muhammad Nasrum Massi, Rufika Shari Abidin, Abd-ElAziem Farouk, Handayani Halik, Gita Vita Soraya, Najdah Hidayah, Rizalinda Sjahril, Irda Handayani, Mohamad Saifudin Hakim, Faris Muhammad Gazali, Vivi Setiawaty, Tri Wibawa

**Affiliations:** 1 Department of Clinical Microbiology, Hasanuddin University, Makassar, South Sulawesi, Indonesia; 2 Microbiology Laboratory, Hasanuddin University Hospital, Makassar, South Sulawesi, Indonesia; 3 Hasanuddin University Medical Research Center Laboratory, Faculty of Medicine, Hasanuddin University, Makassar, South Sulawesi, Indonesia; 4 Department of Biotechnology, Faculty of Science, Taif University, Taif City, Al-Hawiyya, Saudi Arabia; 5 Mega Rezky University, Makassar, South Sulawesi, Indonesia; 6 Department of Biochemistry, Faculty of Medicine, Hasanuddin University, Makassar, South Sulawesi, Indonesia; 7 Clinical Pathology Laboratory, Wahidin Sudirohusodo Hospital, Makassar, South Sulawesi, Indonesia; 8 Department of Microbiology, Faculty of Medicine, Public Health and Nursing, Gadjah Mada University, Yogyakarta, Indonesia; 9 Master Program in Biotechnology, Postgraduate School, Gadjah Mada University, Yogyakarta, Indonesia; 10 National Institute for Health Research and Development, Ministry of Health, Jakarta, Indonesia

**Keywords:** COVID-19, SARS-CoV-2, Whole-genome sequencing, D614G, N439K

## Abstract

**Introduction:**

A global surge in SARS-CoV-2 cases is occurring due to the emergence of new disease variants, and requires continuous adjustment of public health measures. This study aims to continuously monitor and mitigate the impact of SARS-CoV-2 through genomic surveillance, to determine the emergence of variants and their impact on public health.

**Methods:**

Data were collected from 50 full-genome sequences of SARS-CoV-2 isolates from Makassar, South Sulawesi, Indonesia. Mutation and phylogenetic analysis was performed of SARS-CoV-2 from Makassar, South Sulawesi, Indonesia.

**Results:**

Phylogenetic analysis showed that two samples (4%) were of the B.1.319 lineage, while the others (96%) were of the B.1.466.2 lineage. Mutation analysis of the spike (S) protein region showed that the most common mutation was D614G (found in 100% of the sequenced isolates), followed by N439K (98%) and P681R (76%). Several mutations were also identified in other genomes with a high frequency, including P323L (nsp12), Q57H (ns3-orf3a), and T205I (nucleoprotein).

**Conclusion:**

Our findings highlight the importance of continuous genomic surveillance to identify new viral mutations and variants with possible impacts on public health.

## Introduction

The first cases of coronavirus disease 2019 (COVID-19) were detected in Wuhan, China, on 31st December 2019 (Zhou et al., 2020; Oley et al., 2021). It was subsequently confirmed that these cases were due to severe acute respiratory syndrome coronavirus 2 (SARS-CoV-2) (Coronaviridae Study Group of the International Committee on Taxonomy of Viruses, 2020), a respiratory virus that is highly transmissible between humans, and spread rapidly globally. The outbreak was declared a pandemic by the World Health Organization on 30th January 2020 (WHO, 2020b).

Phylogenic studies indicate that the virus first emerged in late November 2019, and has an evolutionary rate of approximately 1.1 × 10^−5^ subs/site/year (Duchene et al., 2020). Compared to other RNA viruses, coronaviruses (including SARS-CoV-2) have proofreading activity encoded by non-structural protein (nsp) 14, which reduces RNA replication errors (Sevajol et al., 2014; Smith et al., 2013; Wu et al., 2020). However, the highly transmissible nature of SARS-CoV-2 increases the likelihood of viral mutations and the rise of new variants in each infected individual.

Furthermore, variants that confer advantageous traits for viral survival (such as higher receptor binding capacity and immune evasion) are more likely to dominate the viral population, as observed in the case of the D614G variant (Korber et al., 2020; Lauring & Hodcroft, 2021). Although this variant was already present in China in sequences collected in late January to early February 2020, the mutation was first reported in the UK in early March 2020 (Korber et al., 2020). By June/July 2020, the D614G mutation was present in 63% of the total submitted sequences worldwide (Koyama, Platt & Parida, 2020). Viruses with the D614G variant have a higher viral load, but are not associated with disease severity (Korber et al., 2020). Over time, D614G diverged, leading to new variants in different parts of the world, including the B.1.1.7, B.1.351, P.1, B.1.617.2, and B.1.1.529 variants from the UK, South Africa, Brazil, India, and South Africa respectively. These variants are currently classified as variants of concern (VOC) by the World Health Organization. VOC are those associated with an observed increase in viral load, moderate immune evasion, and disease severity (WHO, 2021b; Robert Koch Institut, 2021; ECDC, 2022). The emergence of new impactful variants highlights the importance of the containment viral spread, genomic surveillance, and the development of broadly cross-protective vaccines (Walensky, Walke & Fauci, 2021; Abdool Karim & de Oliveira, 2021).

Indonesia announced the first two confirmed cases of COVID-19 on 2nd March 2020 (Alwan, 2007). By 1st December 2021, there were 4,256,687 (278 new within December 2021 alone) confirmed cases, 143,480 (10 new within December 2021 alone) deaths, and 4,104,964 recoveries, from 510 districts across all 34 Indonesian provinces (WHO, 2020a). However, the incidence of disease varies between areas, due to the archipelagic nature of the country and the spread of the virus. Genomic surveillance plays an important role in the containment of the virus, and in mitigating the emergence of new variants. From March 2020 to April 2021, 1,210 SARS-CoV-2 sequences were identified, accounting for 0.073% of the 1,668,360 infections recorded in Indonesia, with a monthly average of 140 days between specimen collection and sequence upload to Global Initiative on Sharing All Influenza Data (GISAID) (Pradipta et al., 2022). By 10th December 2021, a total of 10,094 SARS-CoV-2 genome sequences had been submitted to GISAID from Indonesia (GISAID, 2021).

Makassar is the capital city of the South Sulawesi province, which lies at the center of Indonesia. It has the largest economy in eastern Indonesia, due in particular to the Makassar Megapolitan region at its center, which acts as a busy hub for transportation throughout the country. As of 19th April 2021, a total of 50 SARS-CoV-2 whole-genome sequencing data collected from both the Wahidin Sudirohusodo and Universitas Hasanuddin hospitals had been submitted to GISAID (Ministry of Health of Republic Indonesia, 2021).

Preliminary data evaluation showed that the N439K variant with a D416G background dominated the SARS-CoV-2 viral population in Indonesia (Cahyani et al., 2021). This variant contains an N439K mutation in the receptor-binding motif (RBM), leading to enhanced binding affinity for the human angiotensin I-converting enzyme 2 (hACE2) receptor and resistance to several neutralizing monoclonal and polyclonal antibodies. These properties allow it to infect cells more easily and evade the immune system (Thomson et al., 2021). This study aims to: (1) report the full genome sequences of SARS-CoV-2 isolated from COVID-19 patients in Makassar, South Sulawesi, Indonesia, throughout the period of January to April 2021; and (2) perform mutation and phylogenetic analysis of SARS-CoV-2 from Makassar, South Sulawesi, Indonesia.

## Materials & Methods

### Clinical sample collections

This study was conducted in Makassar, the capital city of the South Sulawesi province of Indonesia. SARS-CoV-2 RNA samples from COVID-19-positive patients were collected at the Wahidin Sudirohusodo and Hasanuddin University Hospitals, which are type A and B COVID-19 referral hospitals, respectively. The participants were recruited using total sampling. SARS-CoV-2 patients were grouped based on the severity of their illness according to the National Institutes of Health, Treatment Guidelines Panel Coronavirus Disease 2019 (COVID-19) as described previously in National Institutes of Health (2021) and El-Raey et al. (2021).

All nasopharyngeal swab samples were collected in viral transport media (Kang Jian Viral Transport Media; Jiangsu Kang Jian Medical Apparatus Co. Ltd., Jiangyan Taizhou, China) and transported to the Hasanuddin University Medical Research Centre (HUM-RC) Laboratory, Faculty of Medicine, Universitas Hasanuddin. SARS-CoV-2 was detected using real-time quantitative PCR (qRT-PCR) with a mBiocov-19 RT-PCR Kit (Biofarma, Bandung, Indonesia) and CFX96 Touch Real-Time PCR Detection System (Bio-Rad, Hercules, CA, USA). For whole-genome sequencing, the samples were selected based on a CT value below 30. Clinical and other laboratory data were obtained from the patient forms, which were sent to the laboratory.

### Full-genome sequencing

The total viral RNA was purified from 50 original nasopharyngeal swab samples using a Viral Nucleic Acid Extraction Kit II (Geneaid Biotech Ltd., New Taipei City, Taiwan). The purified RNA samples were used for whole-genome sequencing (WGS) using the Oxford Nanopore’s GridlON sequencer methodology, operated with MinION version 20.06.9 and MinION core version 4.0.11. High accuracy base calling was conducted using Guppy (Oxford Nanopore Technologies, 2021) version 4.0.11, with all generated reads assembled using the EPI2ME Labs platform employing ARTIC workflow. This provided the whole genome sequences of the collected samples, in addition to data on amino acid mutations in each isolate.

### Genome annotation and phylogenetic analysis

The full-length genomes of the SARS-CoV-2 samples isolated in this study were annotated using the reference genome of hCoV-19/Wuhan/Hu-1/2019 (NC_045512.2). For phylogenetic analysis, a dataset of available SARS-CoV-2 genomes from different countries, including those from other regions in Indonesia, was retrieved from GISAID, representing both VOC and non-VOC. The final dataset consisted of 50 original samples derived from this study, the NCBI reference sequence (NC_045512.2), and 30 sequences retrieved from GISAID, consisting of B.1, B.1.319, B.1.466.2, Alpha (B.1.1.7), Beta (B.1.351), Gamma (P.1), Delta (B.1.617.2), and Omicron (B.1.1.529). The studies from which this data was sourced are provided in Table S1. The accession IDs of our isolates are provided in Table S2.

A total of 81 sequences were aligned using MAFFT (v7.471) with the L-INS-i algorithm (Katoh & Standley, 2013)**.** The aligned dataset was used to construct the maximum likelihood (ML) tree, using RaXML v.8.2.10 with 1,000 bootstraps and the GTR+G model as the best-fitting substitution model (Stamatakis, 2014)**.** The ML tree was viewed and annotated using MEGA (v11.1.1) (Tamura, Stecher & Kumar, 2021). The final tree was rooted in the ancestor virus hCoV-19/Wuhan/Hu-1/2019 to visualize the evolutionary relationship between our original samples and the other SARS-CoV-2 isolates deposited in GISAID.

### Mutation visualization

All mutations were visualized in a 3D protein model using Swiss-PdbViewer (ver. 4.1.0). The protein model was reconstructed using the Wuhan reference sequence (NC_045512.2) against the S Cryo-EM structure (PDB ID: 6XR8; Resolution: 2.90 Å), to observe mutations located at the furin cleavage site.

Protein modelling was performed using the Swiss-Model web server (https://swissmodel.expasy.org/). The quality of the protein model was ensured by observing the QMEAN (>−4.0).

### Ethical approval

This study obtained ethical approval from the Health Research Ethical Committee of the Faculty of Medicine, Universitas Hasanuddin, Makassar, South Sulawesi, Indonesia (No. 845/UN4.6.5.31/PP36/2020). Written informed consent was obtained from all patients prior to participating in this study.

## Results

### Demographics and clinical severity of COVID-19 patients

Over half of the patients in this cohort were male (56%), with the most severely affected patients between the ages of 30–39 years (30%), followed by those aged 50–64 years (20%) and 40–49 years (19%) (see Table 1). Eleven (22%) cases were asymptomatic, while 17 (34%) patients were categorized as mild, 17 (34%) as moderate, and the remainder (10%) as severe. Patients with comorbidities were found in a moderately high proportion (60% of 10 samples) in this study, as shown in Table 1.

### Full genome characterization of SARS-CoV-2 isolated from Makassar, South Sulawesi

**Figure 1:**
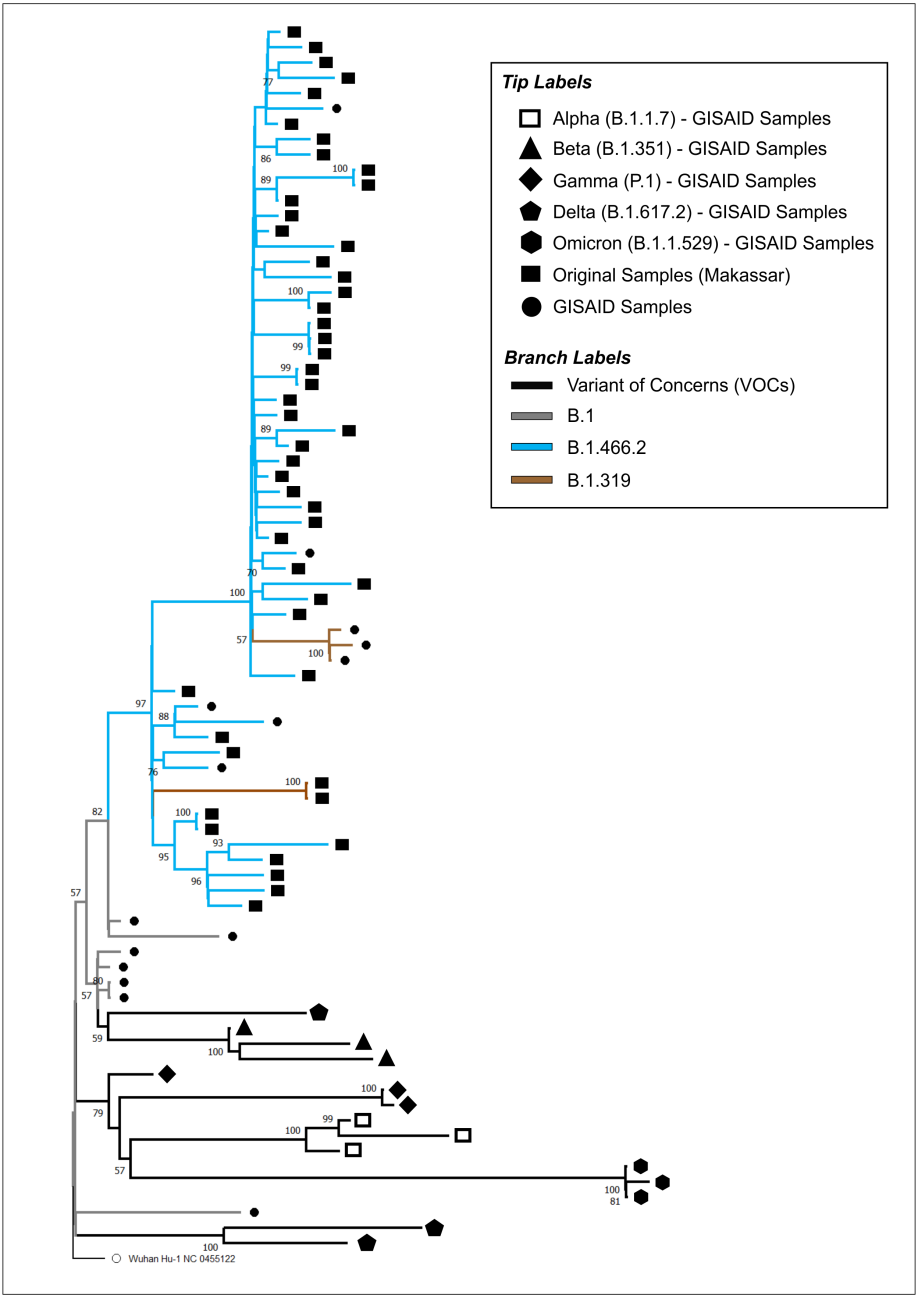
Phylogenetic analysis of SARS-CoV-2 from Makassar and other countries. A phylogenetic tree was constructed from the full-length genome of SARS-CoV-2 using the maximum likelihood statistical method, with 1,0000 bootstrap replications and the best DNA substitution model for the dataset (GTR+G). Virus isolates originating from Makassar are indicated in green. The tree was rooted on the ancestor virus hCoV-19/Wuhan/Hu-1/2019.

The phylogenetic tree of our original isolates (*n* = 50) and GISAID sequences (*n* = 31) is shown in Fig. 1. All isolates originating from Makassar were clustered in the main lineage of B.1, where 48 samples were identified from the B.1.466.2 lineage, and two (hCoV-19/Indonesia/SN-MKS_17/2021 and hCoV-19/Indonesia/SN-MKS_36/2021) from the B.1.319 lineage. However, 38 sequences of our isolates formed new clades which were different from other samples from the GISAID reference lineage of B.1.466.2 (EPI_ISL_877456; EPI_ISL_768689; EPI_ISL_728184). All isolates from Makassar were distantly related to other VOC lineages (Alpha/B.1.1.7, Beta/B.1.351, Gamma/P.1, Delta/B.1.617.2, Omicron/B.1.1.529).

**Table 1:** The clinical characteristics of patients with COVID-19 in Makassar, South Sulawesi, Indonesia, from January to April 2021.

Variable	N (%)
**Gender**	
Male	28 (56)
Female	22 (44)
**Age (year)**	
0–4	4 (8)
5–17	1 (2)
18–29	9 (8)
30–39	15 (30)
40–49	5 (19)
50–64	10 (20)
65–74	4 (8)
75–84	2 (4)
**Clinical severity**	
Asymptomatic	11 (22)
Mild	17 (34)
Moderate	17 (34)
Severe	5 (10)
Comorbidities^*^	
Hypertension	1 (10)
Coronary Artery Disease	1 (10)
AV Block	1 (10)
Coagulopathy	2 (10)
Malnutrition	1 (10)
Elevated Liver Enzyme	1 (10)
Anxiety Disorder	1 (10)

**Notes.**

* Data available in 10 samples.

### Mutation analysis of the S protein

**Figure 2:**
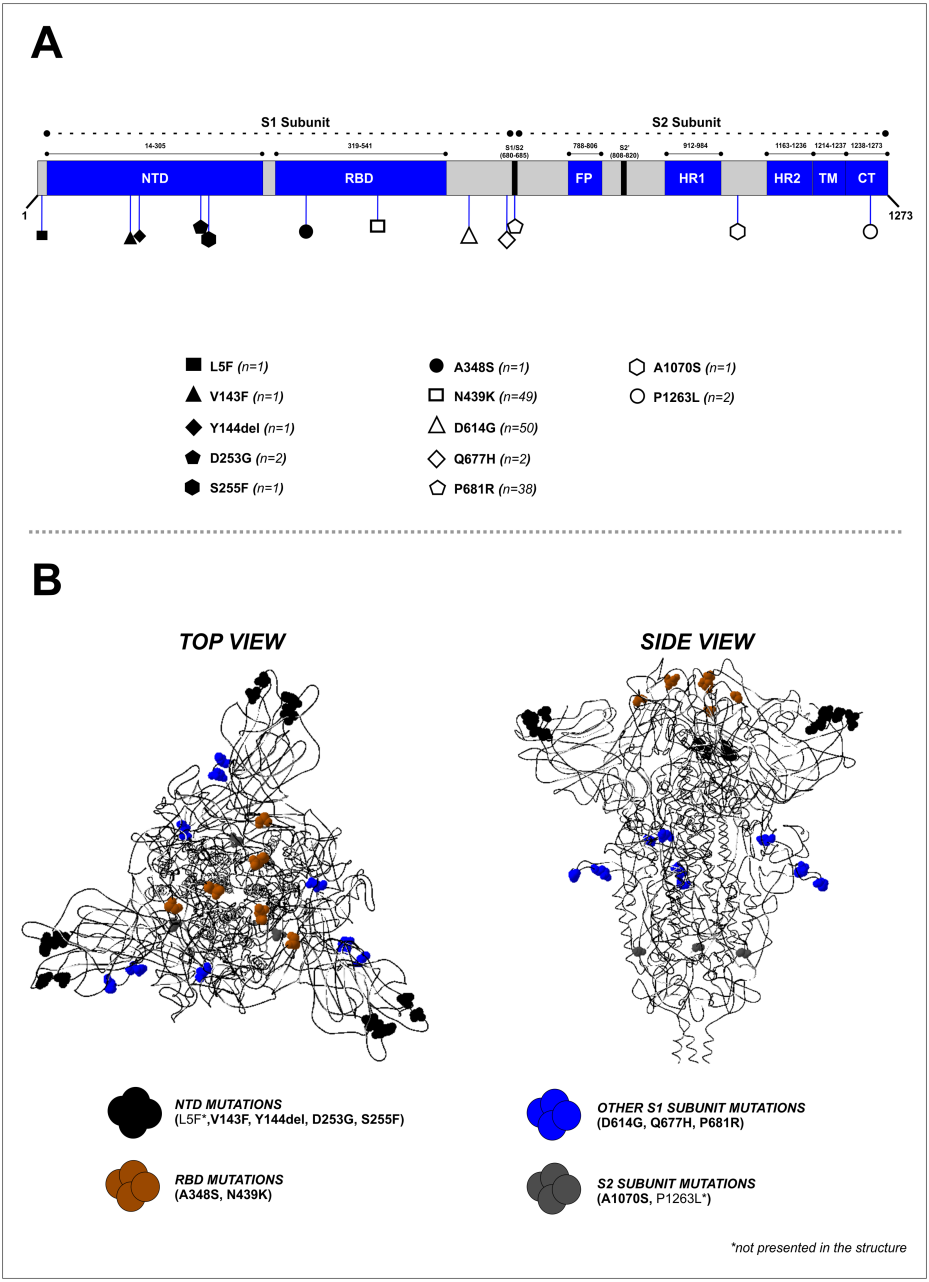
Mapping of the S protein mutations identified in this study. (B) Visualization of the 3D structure of the S protein containing identified mutations, in top and side views. The mutations were found predominantly in the S1 subunit—10 mutations were found in this region, compared to only two in the S2 subunit.

Mutation analysis of the S protein region showed that the most frequently occurring mutations in our study were D614G (50/50, 100%), followed by N439K (49/50, 98%) and P681R (38/50, 76%) (Fig. 2A and Table 2). Other mutations were found at lower frequencies, including L5F, V143F, Y144del, D253G, S255F, A348S, Q677H, A1070S, and P1263L. Mapping of the identified mutations showed they were predominantly in the S1 subunit (10 mutations), while two were located in the S2 subunit, and two others (A348S and N439K) were found in the receptor-binding domain (RBD). D641G, P681R, and Q677H mutations were located close to the furin cleavage site (FCS) region (Fig. 2B). Four mutations (V143F, Y144del, D253G, and S255F) were identified in the N-terminal domain (NTD) of the S1 subunit (Fig. 2A). The distribution of these specific mutations in our collected samples are provided in Table S2.

**Table 2:** The dominant mutations in SARS-CoV-2 proteins identified in this study. Mutations with a frequency of > 10% in our collected samples are included.

No	Region	Mutation	Frequency [n (%)]
1	nsp3-ORF1ab	S126L	38 (76)
		T350I	50 (100)
		P822L	49 (98)
2	nsp6-ORF1ab	L75F	38 (76)
		V149F	6 (12)
3	nsp12-ORF1ab	P323L	50 (100)
4	nsp13-ORF1ab	S259L	38 (76)
5	Spike	N439K	49 (98)
		D614G	50 (100)
		P681R	38 (76)
6	NS3-ORF3a	Q57H	49 (98)
7	Nucleoprotein	T205I	50 (100)

### Mutation analysis of other SARS-CoV-2 genome regions

Mutations occurring with high frequency (>10%) in other regions of the SARS-CoV-2 genome are provided in Table 2 and Table S2. In the structural protein, we found that the T205I mutation of the N protein was present in all collected samples in this study. Only one mutation (2%) occurred in the envelope (E) protein –L73I. Two mutations were found in the membrane (M) protein at low frequency –I24T (2%) and L46F (4%) (Table S2).

In non-structural proteins, three dominant mutations were identified in the NS3 protein of ORF1ab –S126L (76%), T350I (100%), and P822L (98%). In the nsp12 protein of ORF1ab, all samples had a mutation in P323L. The Q57H mutation in the NS3 protein of ORF3a was found in 49 (98%) of our collected samples. No mutations were identified in nsp4, nsp7, nsp9, or nsp10 of the ORF1ab and ORF10 proteins.

## Discussion

The COVID-19 pandemic continues to present a pressing challenge to global health. Despite possessing a proofreading enzyme, SARS-CoV-2 mutates and adapts rapidly, resulting in new variants with enhanced fitness. By 29th March 2022, the World Health Organization had identified a total of five VOCs, including previously circulating VOCs (Alpha, Beta, Gamma) and VOCs in current circulation (Delta and Omicron) (WHO, 2021b). VOCs tend to demonstrate increased transmissibility, and mitigation measures, such as vaccines, therapeutics, diagnostics, and other public or social health measures, are generally less effective for them (Thomson et al., 2021). Although the parental SARS-CoV-2 strains overwhelmingly affected older age groups in particular, our results show that the virus also affects adolescents and young adults in comparable proportions. Global genomic surveillance of SARS-CoV-2 plays an important role in mitigating and monitoring the emergence and spread of new variants, as well as in providing information for public health measures (WHO, 2021; WHO, 2021a).

This study reports SARS-CoV-2 genomic surveillance using whole-genome sequencing in Makassar, the capital city of the South Sulawesi province of Indonesia, from January to April 2021. We performed phylogenetic and mutation analysis for in-depth characterization of the SARS-CoV-2 variants circulating in this region.

Phylogenetic analysis showed that most isolates originated from the B.1.466.2 lineage, while the others (two samples) belonged to the B.1.319 lineage. These results were higher than those of another study carried out in West Java, another province in Indonesia, between September 2020 to June 2021, which found that 27% of isolates were of the B.1.466.2 lineage (Fibriani et al., 2021). B.1.466.2 variants are more locally dispersed in Indonesia, and have been detected at lower rates in other Southeast Asian countries (Cahyani et al., 2021) B.1.466.2 is one of the Indonesian lineages identified by PANGO, and although it has not been considered a cause for concern on a global scale, it has been designated on alert for further monitoring by the World Health Organization (WHO, 2021b).

The results of the mutation analysis show that ORF1ab contains the greatest number of amino acid mutations, followed by the ORF3a, Spike, and N. The ORF1ab combines codes of a polyprotein that are cleaved into 16 NSPs, which play a variety of roles in viral replication (Yoshimoto, 2020). Amino acid mutations are also able to affect the structure of proteins, which can impact their function in viral replication. The ORF1ab consists of two amino acid mutations (T350I and P323L) observed in 100% of the genome sequences in the cohort. The *ORF3a* gene, which encodes an ion channel protein related to NLRP3 inflammasome reactivation (Yoshimoto, 2020), was found to have a total of 16 amino acid mutations in the cohort, with the Q57H mutation present in 49 (98%) sequences. The nucleocapsid (N) protein encoded by the *N* gene encapsulates the RNA genome of SARS-CoV-2 (Yoshimoto, 2020), and all 50 sequences collected in this cohort were found to contain the T205I mutation.

The S protein, encoded by the *S* gene, is a SARS-CoV-2 surface glycoprotein of particular interest, as it plays an essential role in viral entry into the host cell by binding to cell surface angiotensin-converting enzyme 2 (ACE2) proteins. The S protein is a target for antibody neutralization; thus, amino acid mutations in antibody binding sites within the S protein allow for the evasion of antibody-based host defense mechanisms (Walls et al., 2020). In addition, the RNA sequence of the S region is employed as the target of the primer used in the PCR viral detection method. Consequently, nucleic acid mutations in these areas will result in false negative PCR results (Wang et al., 2020; Vogels et al., 2020). All genomic sequences collected in this cohort contain the background D614G (B.1. lineage) mutation, and 98% of them contain the N439K mutation.

The D614G mutation quickly became prominent in SARS-CoV-19 strains (Korber et al., 2020; Zhou et al., 2022; Viedma et al., 2021), and remains present in VOCs circulating currently. D614G was detected in early cases of COVID-19 in several Europe regions, and quickly became dominant (55–85% by the end of April 2020) (Viedma et al., 2021; Tang et al., 2020). In East Asia, the transition from D to G was slower, with almost 90% of viral samples from Chinese patients still displaying D614 at the end of April 2020 (Tang et al., 2020). In Indonesia, D614G was reported to be dominant (94.2%) until June 2021 (Cahyani et al., 2021), and this is consistent with our findings in Makassar city during our study period of January–April 2021. In other Indonesian provinces (namely Yogyakarta and Central Java) the D614G variant circulating since May 2020 finally replaced the D614 variant completely as of September 2020 (Wijayanti et al., 2022). Several studies have attempted to examine the effects of this mutation, and results suggest that it is a crucial to the FCS region. Structural and biochemical research on a full-length G614 spike trimer revealed that there are connections in D614G not seen in D614, which prevent the S1 subunit from prematurely losing its binding to the ACE2 (Zhang et al., 2021). Other reports found that the change of amino acid at position 614 from aspartic acid (D) to glycine (G) results in a greater ability to penetrate ACE2-expressing cells and replicate in human respiratory cells (Plante et al., 2021; Zhang et al., 2020). This ability essentially increases the number of spikes that can allow infection to occur, and indicates that the D614 viral spike is less stable than the G614 variant. The G614 mutation is thought to confer a moderate degree of infectivity and transmissibility, which could account for its persistent circulation worldwide (Korber et al., 2020; Plante et al., 2021; Zhang et al., 2020).

Similarly to B.1.466.2, the N439K mutation was found in moderately high frequency in this study. The N439K mutation was first detected in Scotland in March 2020 and increased in prevalence until June 2020, before disappearing entirely from the country following stringent public health measures. Despite this, the mutation has reappeared independently several times in other parts of the world. It was first sampled in Romania in May 2020, and as of January 2021 had been detected in 34 countries as the second most observed receptor-binding domain (RBD) mutation worldwide (Thomson et al., 2021). The N439K mutation was most dominant in Indonesia between January and April 2021, and formed its own characteristic lineage (B.1.466.2) in the Indonesian population (Rambaut et al., 2020; O’Toole et al., 2021a; O’Toole et al., 2021b), as reflected in the samples obtained for this study. Experiments involving recombinant N439K, hACE2 proteins, and X-ray structural analysis have shown that the N439K mutation results in a 2-fold increase in hACE2 binding affinity (Thomson et al., 2021). The effect of this on transmissibility and infectivity measures requires further evaluation. The clinical outcomes and viral replication of N439K mutants in cell tissue culture were comparable to those of N439 SARS-CoV-2 (Thomson et al., 2021). The N439K mutants demonstrated more significant 2-fold reduction in response to polyclonal antibody in a small proportion (6.8%) of sera collected from 442 individuals who recovered from COVID-19. A reduction in binding of more than 2-fold was also demonstrated in 16.7% of 140 mAbs isolated from recovered individuals (Thomson et al., 2021). In addition, Zhou et al. (2021) found that the N439K RBD was significantly resistant to the SARS-CoV-2 neutralizing antibody REGN10987, which could potentially result in neutralization failure. This information is important for ensuring the efficacy of vaccines and immunotherapy. Amino acid changes appear to accumulate gradually over time. N439K is the most prominent mutation in the RBD domain, and should be used preferentially to determine the effect of mutations on pathogen evolution.

Another amino acid mutation that should be highlighted is found in the spike protein–P681R. The frequency of P681R was found to be moderately high in Makassar during the study period, compared to data from the rest of Indonesia (42.6%) and the world (2.1%). The potential causes of this, including natural conditions, lifestyle, genetic factors of hosts, and adherence to health protocols, should be further investigated. Studies on viruses with P681R have revealed that the mutation could result in a reduction in neutralizing antibodies. Furthermore, it has been shown to increase cell–cell fusion by promoting S protein cleavage, mediated by furin (Saito et al., 2021). Interestingly, P681R was found frequently in the Delta variant, based on data obtained from GISAID from June to August 2021 (92.7% of all data submitted to GISAID and 99.6% of all Delta variants submitted to GISAID). This mutation may have benefitted from a cumulative fitness advantage over other variants, due to its high frequency in the Indonesian B.1.466.2 lineage.

These findings highlight the necessity of ongoing surveillance to identify novel COVID-19 mutations. The ability to detect new variants and their spread is critical for appropriate public health response and policy making.

## Conclusion

We report the full-genome sequences of SARS-CoV-2 collected from Makassar, South Sulawesi, Indonesia, between January and April 2021. Lineage B.1.466.2 represents the most dominant (96%) of our isolates, and none of these variants are classified as VOC. All SARS-CoV-2 isolates were found to contain the D614G mutation, while the N439K mutation was also found in high proportions (98%) compared to the lineage in the total Indonesian SARS-CoV-2 sequences submitted to GISAID. Our study therefore highlights the importance of continuous genomic surveillance of SARS-CoV-2, particularly in the South Sulawesi region, as well as in other parts of Indonesia, to monitor the emergence and spread of novel mutations and variants with possible impacts on global health.

##  Supplemental Information

10.7717/peerj.13522/supp-1 Supplemental Information 1 The acknowledgment table for GISAID

10.7717/peerj.13522/supp-2 Supplemental Information 2 The amino acid variations of 50 SARS-CoV-2 isolates collected from Makassar, South Sulawesi, Indonesia The positions referred to the reference sequence NC_045512.2. The frequencies of each mutation are depicted in the lowest rows.

## Additional Information and Declarations

### Microarray Data Deposition

The following information was supplied regarding the deposition of microarray data:
https://www.epicov.org/epi3/frontend#252fd0

### Data Availability

The following information was supplied regarding data availability: The SARS-CoV-2 genome sequences used in this study are available via GISAID (https://www.epicov.org/epi3/frontend#252fd0): EPI_ISL_1247019; EPI_ISL_1247873; EPI_ISL_1248934; EPI_ISL_1249610; EPI_ISL_1250222; EPI_ISL_1258423; EPI_ISL_1258424; EPI_ISL_1258463; EPI_ISL_1258464; EPI_ISL_1258465; EPI_ISL_1263319; EPI_ISL_1263320; EPI_ISL_1263321; EPI_ISL_1263322; EPI_ISL_1263323; EPI_ISL_1630277; EPI_ISL_1630278; EPI_ISL_1630279; EPI_ISL_1630280; EPI_ISL_1630281; EPI_ISL_2691912; EPI_ISL_2692137; EPI_ISL_2692142; EPI_ISL_2692143; EPI_ISL_2692144; EPI_ISL_2692169; EPI_ISL_2692170; EPI_ISL_2692171; EPI_ISL_2692179; EPI_ISL_2692205; EPI_ISL_2692206; EPI_ISL_2692531; EPI_ISL_2692533; EPI_ISL_2692706; EPI_ISL_2692880; EPI_ISL_2692882; EPI_ISL_2692883; EPI_ISL_2692884; EPI_ISL_2692886; EPI_ISL_2692887; EPI_ISL_2692889; EPI_ISL_2692891; EPI_ISL_2692892; EPI_ISL_2692896; EPI_ISL_2692897; EPI_ISL_2692899; EPI_ISL_2692900; EPI_ISL_2692902; EPI_ISL_2692903; EPI_ISL_2692905.
